# Phosphocyclocreatine is the dominant form of cyclocreatine in control and creatine transporter deficiency patient fibroblasts

**DOI:** 10.1002/prp2.525

**Published:** 2019-12-20

**Authors:** Kirill Gorshkov, Amy Q. Wang, Wei Sun, Ethan Fisher, Marta Frigeni, Marc Singleton, Natasha Thorne, Bradley Class, Wenwei Huang, Nicola Longo, Minh‐Ha T. Do, Elizabeth A. Ottinger, Xin Xu, Wei Zheng

**Affiliations:** ^1^ National Center for Advancing Translational Sciences National Institutes of Health Bethesda MD USA; ^2^ Division of Medical Genetics Department of Pediatrics University of Utah Salt Lake City UT USA; ^3^ Associated Regional and University Pathologists (ARUP) Laboratories Salt Lake City UT USA; ^4^ Lumos Pharma Austin TX USA

**Keywords:** creatine, creatine transporter deficiency, cyclocreatine, HILIC UPLC–MS/MS method, phosphocreatine, phosphocyclocreatine

## Abstract

Creatine transporter deficiency (CTD) is a metabolic disorder resulting in cognitive, motor, and behavioral deficits. Cyclocreatine (cCr), a creatine analog, has been explored as a therapeutic strategy for the treatment of CTD. We developed a rapid, selective, and accurate HILIC ultra‐performance liquid chromatography‐tandem mass spectrometry (UPLC‐MS/MS) method to simultaneously quantify the intracellular concentrations of cCr, creatine (Cr), creatine‐d3 (Cr‐d3), phosphocyclocreatine (pcCr), and phosphocreatine (pCr). Using HILIC‐UPLC‐MS/MS, we measured cCr and Cr‐d3 uptake and their conversion to the phosphorylated forms in primary human control and CTD fibroblasts. Altogether, the data demonstrate that cCr enters cells and its dominant intracellular form is pcCr in both control and CTD patient cells. Therefore, cCr may replace creatine as a therapeutic strategy for the treatment of CTD.

**Abbreviations**
ADP: adenosine diphosphate
AGAT: L‐Arginine:glycine amidinotransferase
ATP: adenosine triphosphate
BSA: bovine serum albumin
cCr: cyclocreatine
Cr: creatine
CT1: Creatine Transporter 1
CTD: Creatine Transporter Deficiency
GAMT: guanidinoacetate‐N‐methyltransferase
GPA: guanidinopropinonic acid
HILIC‐UPLC‐MS/MS: hydrophilic‐interaction ultra‐performance liquid chromatography‐tandem mass spectrometry
LLOQ: lower limit of quantification
pcCr: phosphocyclocreatine
pCr: phosphocreatine
PK: pharmacokinetics
QC: quality control
SRM: selected reaction monitoring
ULOQ: upper limit of quantification
WT: wild‐type

**Highlights**

- We quantitatively and simultaneously measure creatine, cyclocreatine, and their phosphorylated forms in normal and creatine transporter deficiency cell lysates.
- Cyclocreatine is transported inside cells and converted to phosphocyclocreatine in healthy control and CTD fibroblasts.
- Phosphocyclocreatine is the dominant form of cyclocreatine, whereas phosphocreatine is found in a more balanced ratio with creatine.
- Cyclocreatine addition decreases creatine uptake in fibroblasts, suggesting a competitive uptake mechanism in normal cells, and an alternative uptake mechanism in disease cells.

## 1 INTRODUCTION

Creatine transporter deficiency (CTD) is an X‐linked metabolic disorder characterized by cerebral creatine (Cr) deficiency and affects approximately 1% of males diagnosed with non‐syndromic mental disability.^1^ Mutations in the *SLC6A8* gene impair Creatine Transporter 1 (CT1)‐mediated transport of Cr from the blood circulation into cells.^1^ Currently, there are no available therapies for CTD, and supplementation with Cr has not been effective due to the lack of functional CT1 in these patients.

Under normal conditions, creatine enters the bloodstream via digestion of food or enzymatic synthesis in the kidney and liver by L‐Arginine:glycine amidinotransferase (AGAT) (http://www.chem.qmul.ac.uk/iubmb/enzyme/EC2/1/4/1.html) and guanidinoacetate‐N‐methyltransferase (GAMT) (http://www.chem.qmul.ac.uk/iubmb/enzyme/EC2/1/1/2.html) from arginine, glycine, and S‐adenosyl‐methionine. Once Cr enters cells through the CT1, it is phosphorylated in a reversible reaction by creatine kinase (http://www.chem.qmul.ac.uk/iubmb/enzyme/EC2/7/3/2.html) with adenosine triphosphate (ATP) to phosphocreatine (pCr) and adenosine diphosphate (ADP).^2^ The pCr acts as a reservoir buffer to maintain ATP levels in skeletal muscle and neuronal cells, and rapidly supplies energy during periods of high activity when the need for ATP is dramatically increased. In CTD patients, the lack of ATP buffer by the Cr‐pCr pair due to CT1 and intracellular Cr deficiencies is believed to contribute to the disease pathophysiology and neuronal symptoms.

Cyclocreatine (1‐Carboxymethyl‐2‐iminoimidazolidine; cCr), an analog of creatine, is under investigation as a potential treatment for CTD.^3^ In CTD patients, cCr enters cells independently of CT1 and can be similarly converted to phosphocyclocreatine (pcCr), which provides an alternative energy source to replace the ATP‐buffering function of the Cr‐pCr pair. In order to understand the intracellular levels and pharmacokinetics of cCr and pcCr, a sensitive analytical method is needed.^4^, ^5^ Previous literature reports analyzing cyclocreatine using HILIC‐UPLC‐MS/MS methods have been used to determine pharmacokinetics (PK) from rodent plasma^4^ or to analyze dietary supplement components .^6^ However, simultaneous measurements of cCr uptake in CTD patient cells and quantification of pcCr in cell lysate has not yet been reported. Therefore, we developed a sensitive and robust HILIC‐UPLC–MS/MS method for determinations of intracellular Cr, cCr, and their phosphorylated forms in both wild‐type (WT) and CTD patient fibroblasts.

## 2 MATERIALS AND METHODS

### 2.1 Chemicals and reagents

LC‐MS grade acetonitrile (ACN) and methanol (MeOH) were purchased from Fisher Scientific (Pittsburgh, PA). Deionized water was purified by a Milli‐Q Ultrapure Water Purification System from EMD Millipore Corporation (Billerica, MA). Cyclocreatine (cCr), Creatine‐d3 monohydrate (98 atom % D) (Cr‐d3), pcCr, phosphocreatine‐d3 (pcCr‐d3), 3‐Guanidinopropinonic acid (≥99%) (GPA), ammonium acetate and formic acid were purchased from Sigma Aldrich (St. Louis, MO). pcCr and stable‐labeled internal standard (IS), cyclocreatine‐D4 [2‐(2‐imino‐4,4,5,5‐D4‐imidazolidin‐1‐yl)acetic acid] (cCr‐d4), were synthesized in‐house.^6^

### 2.2 Cell culture

WT (GM08333 Fibroblast from Skin, Foreskin; Coriell Institute (Camden, NJ)) and four lines of patient fibroblasts were cultured using CTD media (DMEM + 10% FBS + 1% Penicillin Streptomycin + 1% L‐glutamine + 1% Amphotericin B). After 70%‐85% confluency was achieved, cells were transferred into either ThermoFisher Scientific 150 mm x 20 mm Nunclon Delta Surface cell culture dish (5E5 cells/well) or Corning 6 well cell culture plate (2E5 cells/well) for compound treatment. After overnight incubation at 37°C, 5% CO2, 75% humidity, cells were treated with compound prepared in CTD uptake media (DMEM + 1% FBS + 1% Penicillin/Streptomycin + 1% L‐glutamine) to encourage uptake of compound by the cells. Cells were collected after allotted compound treatment duration.

### 2.3 In vitro uptake experiments

To determine the ability of cCr to achieve therapeutic concentrations in vitro, the following conditions were initially tested in WT and patient line 1 fibroblasts: a 72‐hours dose response assay was run to identify the lowest required concentration to achieve a therapeutic concentration. The same cell lines were exposed to the following conditions: 2.0 mmol/L cCr, 1.0 mmol/L cCr, 500 µmol/L cCr, 250 µmol/L cCr, 125 µM cCr, 25 µmol/L cCr, 25 µmol/L Cr‐d3, and no compound. Lastly, a kinetic study was performed to determine the time required to achieve therapeutic concentrations in vitro. In this experiment, WT and patient line 1 cells were treated with either 500 µmol/L cCr, 500 µmol/L cCr + 1.0 mmol/L GPA, 500 µmol/L Cr, or 500 µmol/L Cr + 1.0 mmol/L GPA for 0 hour, 2.0 hours, 4.0 hours, 24 hours, or 72 hours. The initial results were confirmed in patient lines 2, 3, and 4, treating cells with 500 µmol/L cCr, 500 µmol/L cCr + 1.0 mmol/L GPA, 500 µmol/L Cr, or 500 µmol/L Cr + 1 mmol/L GPA for 72 hours. For all experiments, treatments were performed in triplicate. Following the compound treatment for the allotted times, the cells were harvested for compound analysis by HILIC UPLC‐MS/MS.

### 2.4 Extraction of creatine‐d3, phosphocreatine‐d3, creatine, phosphocreatine, cyclocreatine, and phospho‐cyclocreatine

To measure the levels of Cr‐d3, pCr‐d3, Cr, pCr, cCr, and pcCr in cells, treated cells were isolated and lysed to measure the intracellular compound levels. Prior to harvesting the cells, media and excess compound were removed through two washes with chilled dPBS (‐) (‐). The cells were subsequently trypsinized using TrypLE Express in combination with a 4 minute incubation at 37°C, 5% CO_2_, and 75% humidity. The lifted cells were gathered by washing with chilled PBS (+) (+). The cell mixture was centrifuged at 1600 rpm for 10 minutes at 4°C. The supernatant was removed, and the cell pellet was resuspended with PBS (+) (+) for a final (3rd) wash; cell counting was performed during this stage using LUNA fluorescence cell counter. Once more, the cell mixture was centrifuged at 1600 rpm for 10 minutes at 4°C. The supernatant was removed, and the cell pellet was frozen at −80°C until sonication.

Prior to sonication, the frozen cell pellets were thawed on ice. Once thawed, cells were centrifuged at 1600 rpm for 1 minute. The cell pellet was resuspended with 50 µL Lysis buffer (DPBS (‐) (‐):H_2_O/1:1). Using the Omni Sonic Ruptor 400, the cell mixture was lysed (at 20% Total Power) for five seconds, followed by one minute of rest (on ice) to minimize compound decomposition by overheating. This process was repeated six times for each sample. 15 µL of sonicated cell lysate was removed for protein quantitation. To the remaining 35 µL of sonicated cell lysate, 160 µL precooled methanol was added with one minute of vortexing, followed by 80 µL precooled ultrapure water with three minutes of vortexing. The lysate mixture was spun at 13 200 rpm for 20 minutes and 200 µL supernatant was collected and stored at −80°C for HILIC UPLC‐MS/MS analysis.

### 2.5 Protein quantitation assay (Bradford)

The sonicated cell lysate was centrifuged at 13 200 rpm for 20 minutes at 4°C. During centrifugation, a Greiner white, clear‐bottom 96‐well assay plate was prepared for the Bradford assay. In this plate, 250 µL Bradford reagent were added into each required well—the first column was occupied by 5 µL of standards (ie water, 125 µg/mL BSA, 250 µg/mL BSA, 500 µg/mL BSA, 750 µg/mL BSA, 1000 µg/mL BSA, 1500 µg/mL BSA). The subsequent columns were loaded with 5 µL centrifuged cell lysate supernatant in duplicate. The protein content was determined by measuring the A_595nm_ via the 96 well plate compatible Spectramax M3 spectrophotometer. The mean protein mass and standard deviation were recorded for compound uptake normalization purposes.

### 2.6 HILIC UPLC‐MS/MS analysis

Stock solutions of cCr, Cr, Cr‐d3, pcCr, pCr and cCr‐d4 (IS) at 1.00 mg/mL were prepared by dissolving each compound in water. Calibration standards and quality control samples were prepared in lysate solution. The calibration ranges were 0.5‐1000 ng/mL for cCr, Cr and Cr‐d3; and 10‐10,000 ng/mL for pcCr and pCr. All frozen lysate samples and quality control samples were thawed at room temperature prior to analysis. Once thawed, samples were thoroughly vortexed. In a 2 mL 96‐well plate, a 200 µL aliquot of internal standard working solution (0.2 μmol/L cCr‐d4 in ACN/MeOH:7/3) was added to each well, then a 50 µL aliquot of standards, QCs and samples was added. The 2 mL 96‐well plate was capped, vortexed and centrifuged at 3000 rpm for 20 minutes at 4°C. 150 µL of supernatant was transferred to a 350‐µL 96‐well plate using a TECAN Freedom Evo 200 robotic system (Morrisville, NC) and 1.0 μL of supernatant was injected for HILIC‐UPLC‐MS/MS analysis. Sample plates were kept in the autosampler at 8°C during sample analysis.

HILIC‐UPLC separation was carried out using a Waters Acquity I‐class system (Waters Corp., Milford, MA). The HILIC column was a Waters Acquity BEH amide (1.7 μm, 2.1 × 50 mm) and was maintained at 60°C. The mobile phases were 10 mmol/L ammonium bicarbonate in 5% ACN/H_2_O (v/v) pH 9.2 (A) and 10 mmol/L ammonium bicarbonate in 95% ACN/H_2_O (v/v) pH 9.2 (B). The flow rate was 0.5 mL/min. The optimal UPLC elution gradient was: 0‐0.2 minutes 95% B; 0.2‐1.5 minutes 95 → 50% B; 1.5‐1.8 minutes 95 → 10% B; and 1.8‐2.0 minutes 95% B.

A Waters Xevo TQ‐S triple quadrupole mass spectrometer was operated in positive electrospray ionization (+ESI) mode for all compounds. Mass spectrometric conditions were optimized through infusion of each compound at 5 μL/min. Under these conditions, cCr, Cr, Cr‐d3, pCr, pCr and cCr‐d4 (IS) yielded predominantly protonated ions at m/z 144, 132, 135, 224, 212 and m/z 148, respectively. Each of the precursor ions was subjected to collision‐induced dissociation (CID) to generate product ions. The product ions of cCr, Cr, Cr‐d3, pcCr, pCr and cCr‐d4 (IS) at m/z 98, 90, 93, 98, 90 and 102 were chosen for the selected reaction monitoring (SRM). Experimental parameters were optimized as follows: 150 L/h cone gas, 1000 L/h desolvation gas, 150°C source temperature, 500°C desolvation temperature, 30 V cone voltage and 1 kV of capillary voltage. The collision gas argon was pressurized at 5.5 × 10^−5^ Torr. The optimized collision energies (CE) for cyclocreatine, creatine, creatine‐d3, phosphocyclocreatine and phosphocreatine were 20, 18, 18, 32 and 20 V, respectively. The optimized phosphocreatine SRM parameters were used to quantify phosphocreatine‐d3 with SRM of 215‐93. The results were analyzed by 1/x^2^ weighted least‐squares linear regression using TargetLynx (Waters Corp., Milford, MA).

### 2.7 Alamar blue assay

Each of the five fibroblast lines were seeded into a Greiner black, clear bottom, tissue culture 384 well assay plate at 2000 cells/well in CTD media. The plates were incubated overnight at 37°C (5% CO_2_, 75% humidity). After overnight cell adherence, the cells were treated with 20 mmol/L CCr, 2.0 mmol/L cCr, 200 µmol/L cCr, 20 mmol/L Cr, 2.0 mmol/L Cr, 200 µmol/L Cr, or no compound (in CTD Uptake Media) for 72 hours. One hour prior to the addition of Alamar Blue detection reagent, 20 mmol/L Mefloquine was added to several vehicle control wells to encourage cell death. After 72‐hour incubation, 20 µL 2X Alamar blue was added into each well. The plate was incubated for an additional hour. The metabolic activity associated with each compound treatment was detected using the Envision plate reader.

### 2.8 ATP content assay

Each of the five fibroblast lines were seeded into a Greiner black, clear bottom, tissue culture 384 well assay plate at 2000 cells/well in CTD media. The plates were incubated overnight at 37°C (5% CO_2_, 75% humidity). After proper cell adhesion, the cells were treated with 20 mmol/L cCr, 2.0 mmol/L cCr, 200 µmol/L cCr, 20 mmol/L Cr, 2.0 mmol/L Cr, 200 µmol/L Cr, or no compound (in CTD Uptake Media) for 72 hours. One hour prior to the addition of ATPlite detection reagent, 20 mmol/L Mefloquine was added to several vehicle control wells to encourage cell death. After 72‐hours incubation, 10 µL ATPlite was dispensed into each well and the plates were incubated at room temperature for 10 minutes. Subsequently, the ATP content associated with each compound treatment was detected using the luminescence function provided by the Envision plate reader.

### 2.9 Statistical analysis

Wells in plates were randomly assigned to the various treatments. The data presented for all in vitro experiments were gathered from three independent experiments unless otherwise indicated in the figure caption. Experiments where n < 3 were due to technical difficulties. Microsoft Excel was used to arrange the data and Graphpad Prism was used to generate graphs and for statistical analysis. Non‐linear regression curve fitting was used to generate curves. Two‐way ANOVA with Sidak's multiple comparison test was used to compare mean values. Data presented as mean ± standard deviation.

## 3 RESULTS

### 3.1 HILIC UPLC‐MS/MS bioanalytical method development to measure Cr and its analogs

**Figure 1:**
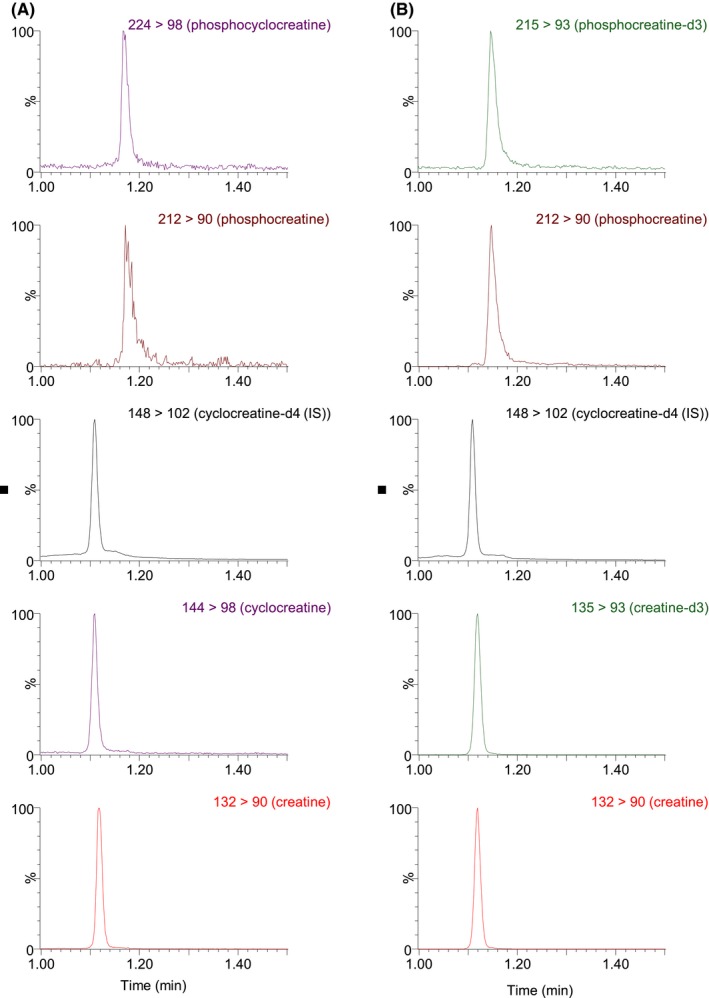
Representative Chromatograms of cell lysate samples: (A) cCr treated sample: pcCr, pCr, cCr‐d4 (IS), cCr and Cr (in a vertical order); (B) Cr‐d3 treated sample: pCr‐d3, pCr, cCr‐d4 (IS), Cr‐d3 and Cr (in a vertical order)

We developed and optimized a HILIC‐UPLC‐MS/MS method to quantify Cr, creatine‐d3 (Cr‐d3), pCr, pCr‐d3, cCr, and pcCr in complex cellular lysates. To measure the Cr uptake and compare the conversion between Cr and cCr, we used the d3 labeled creatine to differentiate it from the endogenous Cr cCr‐d4 was used as the internal standard. We first tested mobile phases with different volatile buffers. A mobile phase with 10 mmol/L ammonium bicarbonate in 5% acetonitrile/water (eluent A) and 10 mmol/L ammonium bicarbonate in 95% acetonitrile/water (eluent B) at pH 9.2 showed the best overall cCr, Cr‐d3, pCr, and pcCr retentions and peak shapes (Figure 1). The peak width was approximately 2.0 seconds, which provided high chromatographic resolution.

Tandem mass spectrometry (MS/MS) parameters were optimized for all analytes to achieve appropriate selectivity and sensitivity. The protonated molecular ions of Cr, Cr‐d3, cCr, cCr‐d4 pCr, and pcCr were all observed in the ESI‐MS positive ionization mode. The Cr, Cr‐d3, cCr, cCr‐d4 pCr, and pcCr precursor ions were fragmented in the collision cell and yielded the most abundant product ions m/z 90, 93, 98, 102, 90, and 98, respectively. This allowed the use of selected reaction monitoring (SRM) for Cr, Cr‐d3, cCr, cCr‐d4, pCr, and pcCr at m/z 132 → 90, 135 → 93, 144 → 98, 148 → 102, 212 → 90, 224 → 98, respectively. Since pCr‐d3 was not available as the authentic analytical standard, SRM of m/z 215 → 93 was monitored to calculate pCr‐d3 concentrations using the pCr calibration standard curve. The optimized HILIC‐UPLC‐MS/MS method offers improved chromatographic resolution and decreased analysis time. The analytical run time is 2.0 minutes. Representative chromatograms of two cell lysate samples are depicted in Figure 1.

The calibration range was 0.5 ng/mL (LLOQ, lower limit of quantitation) to 1000 ng/mL (ULOQ, upper limit of quantitation) for Cr‐d3, Cr, cCr, and 10‐10000 ng/mL for pcCr and pCr. A linear regression with 1/x2 weighting was used to construct the calibration curve. Linear regression of standard curves had slopes of greater than 0.98. The accuracy and precision of quality control (QC) samples (n = 6 at each QC concentration) were summarized in Table 1. Percent recovery of samples was determined to be 83%‐100%. Blank samples were injected in between high‐concentration QC samples to monitor the carryover effect.^7^ There was no observable carryover effect under the experimental conditions used. Together, the results indicated a robust and sensitive HILIC‐UPLC‐MS/MS method for measurements of Cr, pCr, Cr‐d3, pCr‐d3, cCr, and pcCr in samples of cell lysates.

**Table 1:** Assay accuracy and precision (%CV) of LLOQ, LQC, MQC, and HQC samples for cyclocreatine, creatine, creatine‐d3, phosphocyclocreatine, and phosphocreatine

Analyte	n = 6	LLQC (0.5 ng/mL)	LQC (5 ng/mL)	MQC (50 ng/mL)	HQC (1000 ng/mL)
Cyclocreatine (cCr)	Mean (ng/mL)	0.485	5.04	51.6	992
	Accuracy (%)	97	101	103	99
	Precision (%CV)	10.6	2.7	3.2	5.4
Creatine (Cr)	Mean (ng/mL)	0.531	5.38	52.0	1010
	Accuracy (%)	106	108	104	101
	Precision (%CV)	3.9	7.8	3.0	5.1
Creatine‐d3 (Cr‐d3)	Mean (ng/mL)	0.506	5.27	51.3	968
	Accuracy (%)	101	105	103	97
	Precision (%CV)	6.8	4.0	3.5	4.9

Analyte		LLQC (10 ng/mL)	LQC (100 ng/mL)	MQC (1000 ng/mL)	HQC (10 000 ng/mL)
Phosphocyclocreatine (pcCr)	Mean (ng/mL)	10.0	92.0	1020	10 200
	Accuracy (%)	100	92	102	102
	Precision (%CV)	15	6.0	4.3	3.9
Phosphocreatine (pCr)	Mean (ng/mL)	10.1	100	1010	9580
	Accuracy (%)	101	100	101	96
	Precision (%CV)	4.2	9.1	6.2	7.6

### 3.2 Establishing the concentration of cCr in CTD patient cells

Successfully mimicking the functions of naturally occurring Cr with cCr is largely dependent on two actions: uptake of cCr into cells and its phosphorylation to pcCr by creatine kinase to act as a buffer for ATP. To understand if cCr can be used as a substitute to replace Cr, several experiments were performed with WT and CTD patient fibroblast cell lines.

**Figure 2:**
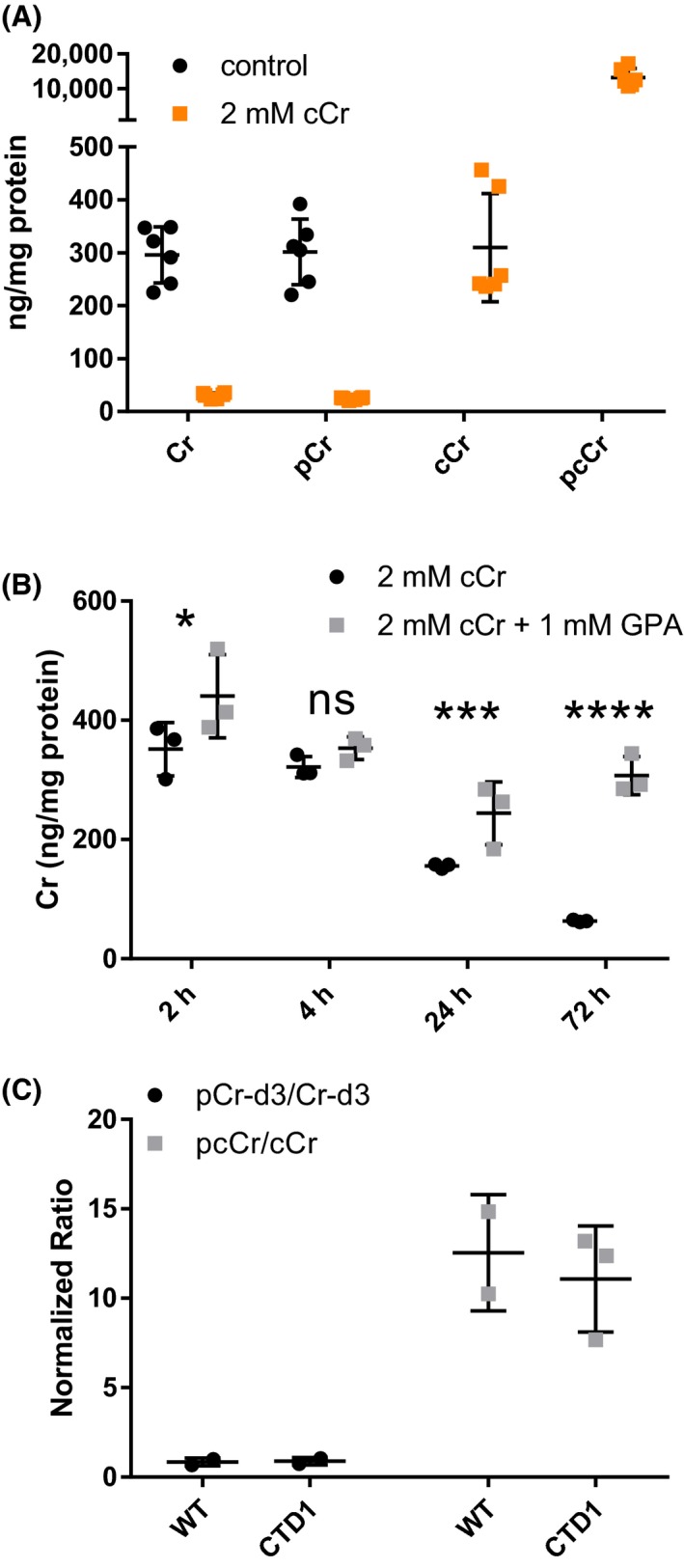
cCr decreases Cr levels in WT fibroblasts. (A) UPLC‐MS/MS quantitative measurements of Cr, pCr, cCr, and pcCr in WT fibroblasts after treatment with 2 mmol/L cCr or vehicle control. (B) Cr concentration in WT cells treated with 2 mmol/L cCr or 2 mmol/L cCr with 1 mmol/L GPA. (C) Ratio of pCr‐d3 to Cr‐d3 and pcCr to cCr concentrations in WT (n = 2) and CTD patient (n = 3) fibroblasts following treatment with 2 mmol/L Cr‐d3 and cCr. Measurements normalized to total protein. Data shown as mean ± SD *indicates *P* < .05, ***indicates *P* < .001, ****indicates *P* < .0001 as calculated by Two‐way ANOVA and Sidak's multiple comparison test. ns, not significant

In WT cells treated with 2 mmol/L cCr for 72 hours at 37°C, the cellular cCr level was 310 ng/mg of total protein while pcCr was 43‐fold higher at 13 200 ng/mg protein. In the same sample, the Cr and pCr levels were 30.0 and 24.4 ng/mg protein, respectively (Figure 2A). Compared to the cCr treated WT cells, the endogenous Cr and pCr levels were 10 fold higher in the WT cells without cCr treatment (Cr, 296 ng/mg; pCr, 302 ng/mg) (Figure 2A). Inhibition of CT1 transport using 1 mmol/L guanidinopropionic acid (GPA), a creatine monohydrate analogue that is the competitive inhibitor of creatine uptake and creatine kinase,^8^, ^9^, ^10^ prevented the cCr‐induced reduction of Cr in WT cells (Figure 2B). This data indicated that cCr competes with Cr for uptake into cells by CT1.^11^

When WT and CTD cells were treated with 2 mmol/L Cr‐d3 or 2 mmol/L cCr, we found that the concentration of pcCr was approximately 10‐fold higher than that of cCr, compared to the approximately 1:1 ratio of pCr‐d3 to Cr‐d3 in both cell lines (Figure 2C). The data indicated that cCr predominately exists in the phosphorylated form (pcCr) compared to Cr‐d3 in both WT and CTD cells which is in an approximately 1:1 ratio (pCr/Cr).

**Figure 3:**
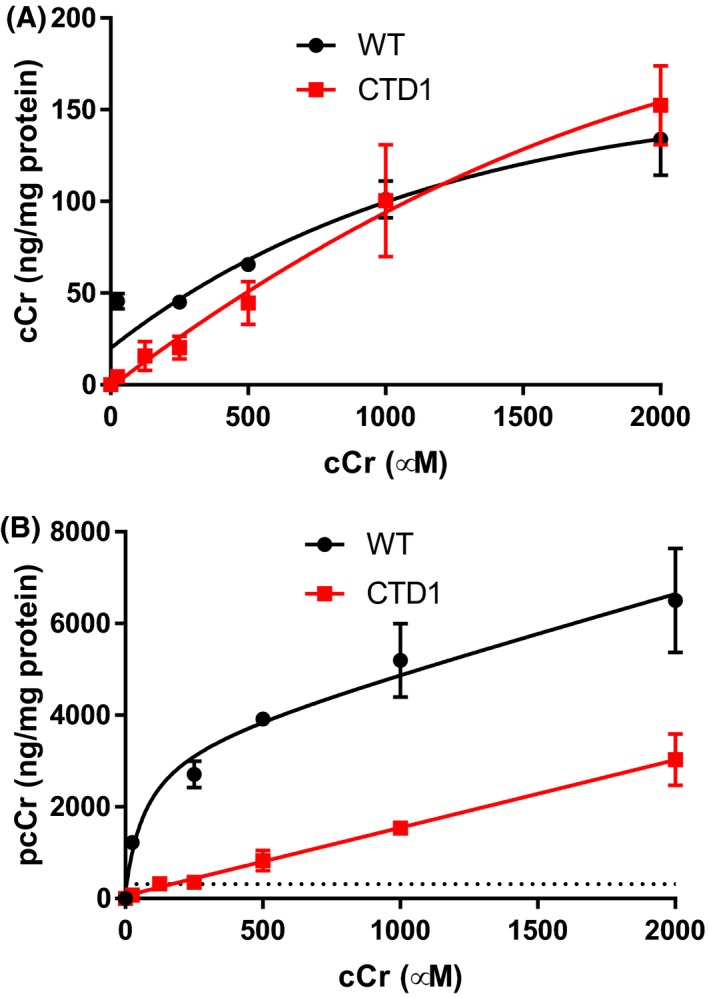
cCr uptake increases with increasing concentrations of cCr (A) After 72 h, cCr concentration‐dependent increases in pcCr in WT and CTD patient fibroblasts. Dotted line indicates concentration of cCr (24.9 µg/mL or 174 µmol/L) required to achieve endogenous concentration of pcCr (318.7 ng/mL). (B) After 72 h, cCr concentration‐dependent increases in cCr in WT and CTD patient fibroblasts. Measurements normalized to total protein. Data fit using non‐linear regression

Next, we investigated the cCr dose required to achieve the previously measured WT Cr concentrations of 300 ng/mg in CTD fibroblast using a six‐point concentration‐response experiment. In patient line‐1 cells (CTD1), 125 µmol/L cCr resulted in a pcCr level of 322 ng/mg of total protein (Figure 3A). As a result, 500 µmol/L cCr treatment was used in the subsequent experiments to ensure a concentration equal to at least the endogenous Cr level of 300 ng/mg of total protein. At equal concentrations of cCr, WT fibroblasts converted cCr to pcCr more readily than CTD patient fibroblasts. CTD fibroblasts exhibited lower cCr concentrations than WT fibroblasts at lower doses of cCr, but intracellular concentrations were similar at doses beyond 1 mmol/L (Figure 3B).

### 3.3 cCr uptake and conversion to pcCr are independent of CT1 in CTD patient cells

**Figure 4:**
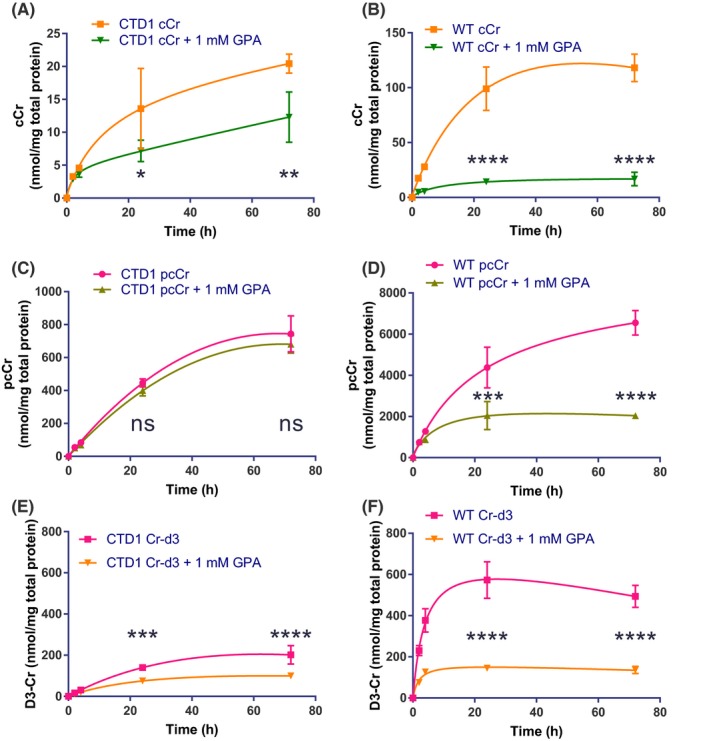
The effect of GPA on the rate of cCr and Cr‐d3 uptake in CTD and WT fibroblasts. CTD patient (A) and WT (B) fibroblast concentration over time curves for cCr in the presence and absence of GPA. CTD patient (C) and WT (D) fibroblast concentration over time curves for pcCr in the presence and absence of GPA. CTD patient (E) and WT (F) fibroblast concentration over time curves for Cr‐d3 in the presence and absence of GPA. Measurements normalized to total protein. Data fit using non‐linear regression. * indicates *P* < .05, ** indicates *P* < .01, *** indicates *P* < .001, **** indicates *P* < .0001 as calculated by Two‐way ANOVA and Sidak's multiple comparison test. ns, not significant

To measure the rate of cCr uptake and cellular retention, WT and CTD patient cells were treated with 500 µmol/L cCr and 500 µmol/L Cr‐d3 with or without the addition of 1 mmol/L GPA (CT1 inhibitor). The cells were harvested at various times to study the cellular and phosphorylated compound levels (Figure 4). It is important to note that the cCr measured in these studies is unphosphorylated cCr, while pcCr is phosphorylated. Total cCr is the sum of unphosphorylated and phosphorylated cCr. The results showed that the difference in cCr uptake of GPA‐treated CTD patient‐line 1 cells was statistically significant. However, the majority of cCr taken up by the cell was converted to pcCr, and therefore the effect of GPA in disease cells may not be physiologically significant (Figure 4A,C). In WT cells, GPA significantly reduced cCr uptake down to disease levels (Figure 4B). The rate of conversion of cCr to pcCr in CTD patient cells was not affected by the addition of GPA (Figure 4C). The levels of pcCr plateaued after 72 hours based on the slope of the non‐linear regression curve fit (Figure 4C,D). In WT cells, GPA treatment reduced the uptake of cCr, thereby reducing the amount of pcCr generated by 60% (Figure 4D). However, the estimated rate of conversion from cCr to pcCr is likely unaffected. The uptake of Cr‐d3 was decreased by the addition of GPA in both CTD and WT fibroblasts, confirming the inhibition of Cr‐d3 uptake by GPA (Figure 4E,F). The concentration of Cr‐d3 was decreased by 50% in CTD patients and by 60% in WT cells. However, the overall concentration of Cr‐d3 in WT cells was two‐fold higher than that of CTD cells after 72 hours without GPA treatment. These experiments revealed disease‐related deficiencies in Cr‐d3 and cCr uptake.

**Figure 5:**
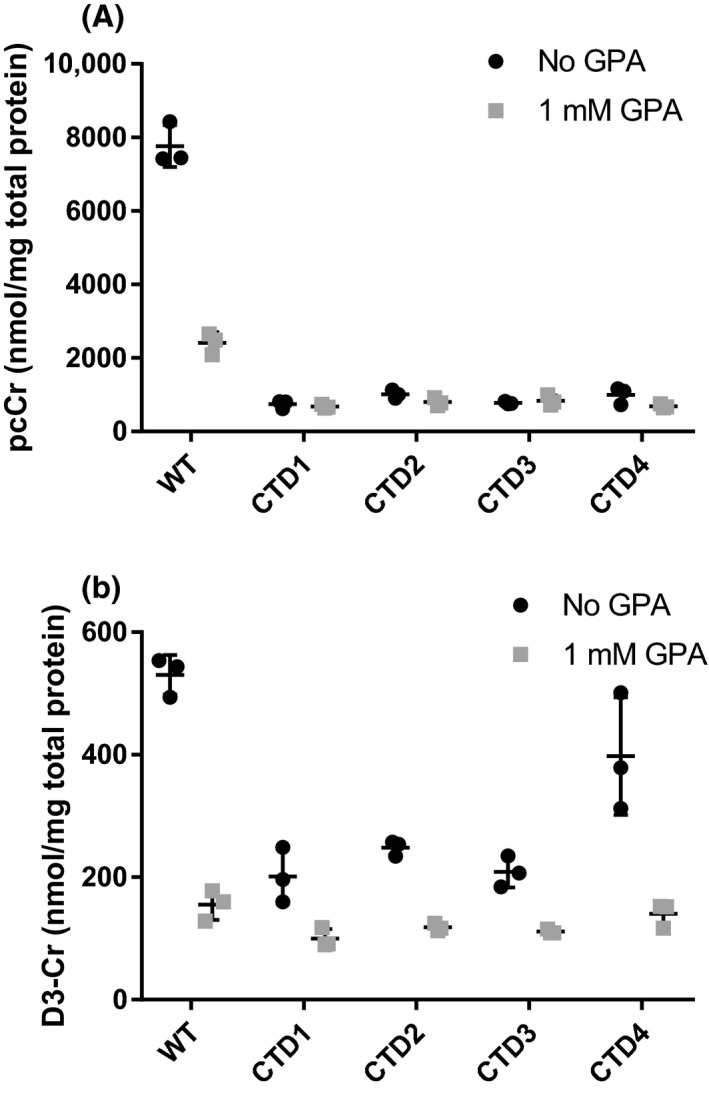
GPA reduces cCr uptake only in WT cells and Cr‐d3 uptake in WT and patient cells. (A) Measured pcCr levels in WT and four different CTD patient fibroblasts treated with cCr in the presence or absence of 1 mmol/L GPA. (B) Measured Cr‐d3 levels in WT and CTD patient fibroblasts treated with cCr in the presence or absence of 1 mmol/L GPA. Measurements normalized to total protein

To confirm this finding, three separate CTD patient lines were treated with 500 µmol/L cCr and Cr‐d3 with and without GPA for 72 hours (Figure 5A,B). In the three additional patient lines, we found that the uptake of cCr and its conversion to pcCr was not affected by GPA, while the uptake of Cr‐d3 was reduced. WT cells exhibited decreased pcCr and Cr‐d3 concentrations in the presence of GPA. Together, the results indicate CTD patient cells exhibit decreased cCr uptake and GPA inhibition of CT1 was less effective than in WT cells (Figure 5). Nonetheless, cCr and pcCr were detected in CTD patient cells. This data provides the therapeutic basis for the use of cCr to treat CTD patients who have a deficiency in CT1 function.

### 3.4 cCr reduced intracellular ATP concentrations without metabolic cytotoxicity

**Figure 6:**
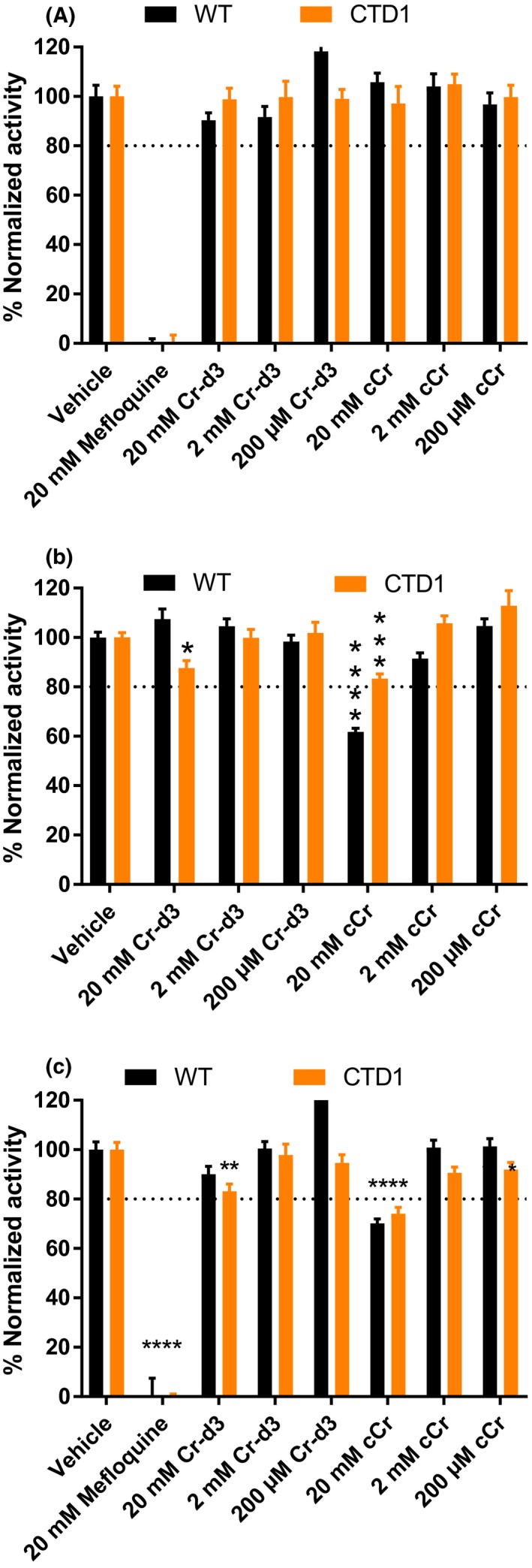
High concentrations of cyclocreatine reduce ATP levels in WT and CTD patient fibroblasts. (A) WT and CTD patient fibroblasts were treated with 20 mmol/L, 2 mmol/L, and 200 µmol/L Cr‐d3 or cCr, vehicle, and 20 mmol/L mefloquine for 72 h and cell viability was measured using Alamar blue. (B) WT and CTD patient fibroblasts were treated with 20 mmol/L, 2 mmol/L, and 200 µmol/L Cr‐d3 or cCr, and vehicle, for 72 h and ATP levels were measured using the ATPlite luminescence assay. (C) WT and CTD patient fibroblasts were treated with 20 mmol/L, 2 mmol/L, and 200 µmol/L Cr‐d3 or cCr, vehicle, and 20 mmol/L mefloquine for 72 h and ATP levels were measured using the ATPlite luminescence assay (replicate of b). Data normalized to vehicle as 100%. Data shown as mean ± SD n ≥ 10 technical replicates. *indicates *P* < .05, **indicates *P* < .01, ***indicates *P* < .001, ****indicates *P* < .0001 as calculated by Two‐way ANOVA and Sidak's multiple comparison test. ns, not significant

The potential cytotoxicity of cCr was measured using two assays including an Alamar Blue cell viability assay that detects metabolic activity and an ATP content cell viability assay that measures ATP levels in live cells. Fibroblasts were treated with 0.2, 2.0, and 20 mmol/L concentrations of cCr and Cr with mefloquine as a positive control compound. After a 72‐hours treatment, no cytotoxicity was observed in either WT or CTD patient fibroblasts treated with cCr or Cr at 0.2 and 2.0 mmol/L concentrations (Figure 6). However, the ATP content assay revealed significant ATP depletion after 20 mmol/L cCr treatment in both WT and CTD cells, as well as a 20% reduction of ATP in 20 mmol/L Cr‐d3 treated cells. Similar results were obtained in the other three patient fibroblast lines (Figure S1). Altogether, the results indicated that at the 500 mol/L cCr dose required to achieve the similar cellular levels of cCr and pcCr to Cr and pCr, cCr treatment had no metabolic toxicity nor observable decrease in cellular ATP levels.

**Figure 7:**
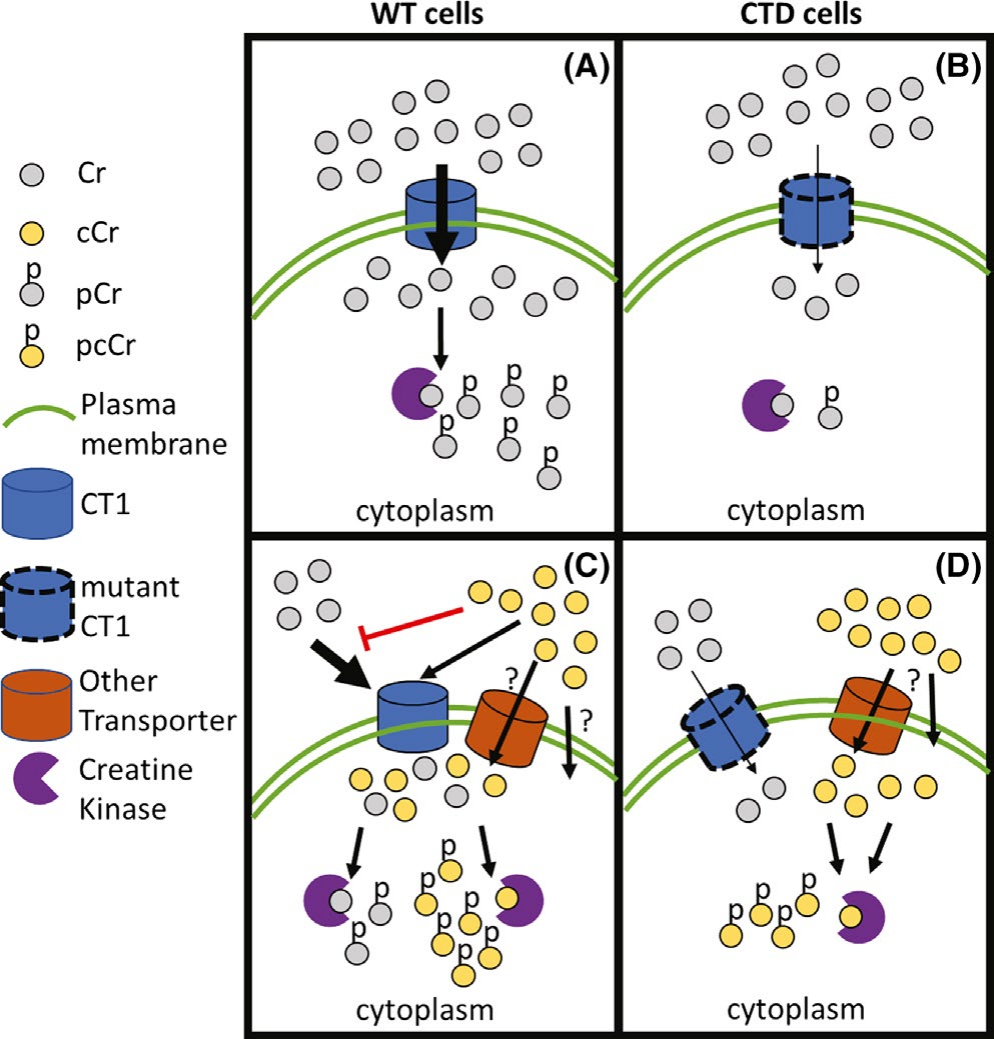
Model of Cr and cCr entry into cells. (A) In WT cells, Cr can enter the cell via CT1 and be phosphorylated by creatine kinase. (B) In CTD cells, CT1 is mutated and dysfunctional, decreasing Cr concentrations in the cell. (C) cCr competes with Cr for cell entry in WT cells with normal CT1 and reduces intracellular Cr concentrations. (D) Thus, another transporter or passive diffusion may mediate cCr entry in CTD cells, providing the necessary cCr‐pcCr pair to buffer ATP levels

In summary, we developed a model to illustrate the entry of Cr and cCr into cells and their conversion to phosphorylated forms (Figure 7). In WT cells, Cr enters the cell via CT1 and be phosphorylated by creatine kinase. In CTD cells, CT1 is dysfunctional, decreasing Cr entry into cells from the extracellular space. cCr competes for cell entry with Cr and also enters cells through an as of yet unknown mechanism, presumably another unknown transporter.

## 4 DISCUSSION AND CONCLUSIONS

cCr is currently under clinical investigation as a viable therapeutic solution for patients with CTD. In this work, we developed a robust and reproducible HILIC UPLC‐MS/MS method to characterize Cr and cCr uptake and the intracellular concentrations of their phosphorylated forms in human normal and CTD patient fibroblasts. The major function of Cr is to act as a buffer to maintain appropriate ATP levels in skeletal muscle and the brain. Due to the CT1 functional deficiency in CTD patient cells, cellular Cr levels are significantly reduced that is linked to disease pathophysiology. cCr supplementation to CTD patients may ameliorate the symptoms by providing a functionally equivalent ATP buffer in patient cells. The Cr‐d3 is used in this study because creatine is present in the cell culture media supplied by the fetal bovine serum.^12^ Furthermore, GAMT and AGAT activity in skin fibroblasts, often used as a diagnostic for associated creatine disorders, leads to endogenous creatine synthesis.^13^, ^14^

Using our HILIC UPLC‐MS/MS method, we are able to simultaneously quantify Cr, cCr, and their phosphorylated species in a single sample. Here, we have determined that cCr is readily taken up by cells and converted to pcCr. We found that the ratio of pcCr to cCr is much higher than that of pCr‐d3 to Cr‐d3 when cells were treated with either cCr or Cr‐d3, respectively. Previous work using 31P‐NMR similarly determined that cyclocreatine is more readily phosphorylated by rabbit muscle creatine kinase.^15^ The differences in the concentration ratios of the phosphorylated form to the unphosphorylated form could possibly be due to either a decreased rate of conversion of Cr‐d3 to pCr‐d3 or increased rate of conversion of pCr‐d3 to Cr‐d3. Similarly, it could be due to an increased rate of conversion of cCr to pcCr or decreased reverse reaction. The results indicate that pcCr could replace the reduced Cr‐pCr pairs in CTD patient cells to buffer ATP levels. In accordance with the observation above, there was more conversion of cCr to pcCr in WT compared to CTD patient fibroblasts with similar levels of cCr uptake, suggesting cCr is more readily phosphorylated by creatine kinase in WT cells compared to CTD fibroblasts. Alternatively, the higher levels of Cr may impact the competition and phosphorylation rate of cCr by creatine kinase.

The expression of CT1^16^ and creatine kinase^17^ in human WT fibroblasts has been confirmed, and overexpression of WT CT1 also restores Cr transport in CTD patient fibroblasts.^18^ We conducted competition experiments with the CT1 transporter inhibitor GPA to understand the uptake mechanism of cCr. While GPA did reduce the uptake of cCr in CTD patient fibroblasts by less than two‐fold, WT fibroblast cCr uptake was significantly impaired in the presence of GPA by more than four‐fold. These results suggest that cCr is preferably transported by the CT1 when it is expressed in cells, and in CTD patient cells cCr may enter through other less efficient mechanisms such as another unknown transporter(s) that are not associated with CT1 transport. Furthermore, while GPA led to a three‐fold reduction of pcCr in WT cells, CTD cells were unaffected, suggesting that GPA’s major role in this system is to inhibit CT1 transport.

The preclinical investigation of cCr as a potential therapy for CTD patients conducted in this work supplies much needed methodology and rich information for cCr uptake in both normal and patient cells. Our work using the HILIC UPLC‐MS/MS analysis of samples from WT and CTD patient fibroblasts has established the needed parameters for evaluation of cCr levels at physiologically relevant doses. Future in vivo studies utilizing this method will provide additional information to guide the cCr clinical studies for CTD.

## DISCLOSURES

Minh‐Ha T. Do is employed by Lumos Pharma.

## AUTHOR CONTRIBUTIONS

AQW, WS, EF, M.S, NT, and BC performed the experiments. WH synthesized reagents. KG, AQW, EF, NT, NL, EAO, WS, XX and WZ interpreted the results. KG, AQW, EF, and WS wrote the manuscript. KG, AQW, WS and EF prepared the figures. KG, EAO, MH.TD and AQW edited the manuscript. XX and WZ supervised all aspects of the work. NT, AQW, XX, and WZ conceived of the original idea for the manuscript.

## Data Availability

Data is available upon request
