# Supplementary material for: Phosphocyclocreatine is the dominant form of cyclocreatine in control and creatine transporter deficiency patient fibroblasts
Source: Pharmacol Res Perspect. 2019 Dec 20;7(6):e00525. doi: 10.1002/prp2.525 (PMC6924099; doi:10.1002/prp2.525)
Supplement: Supplementary file 1 [file PRP2-7-e00525-s001.docx]

**APPENDIX**


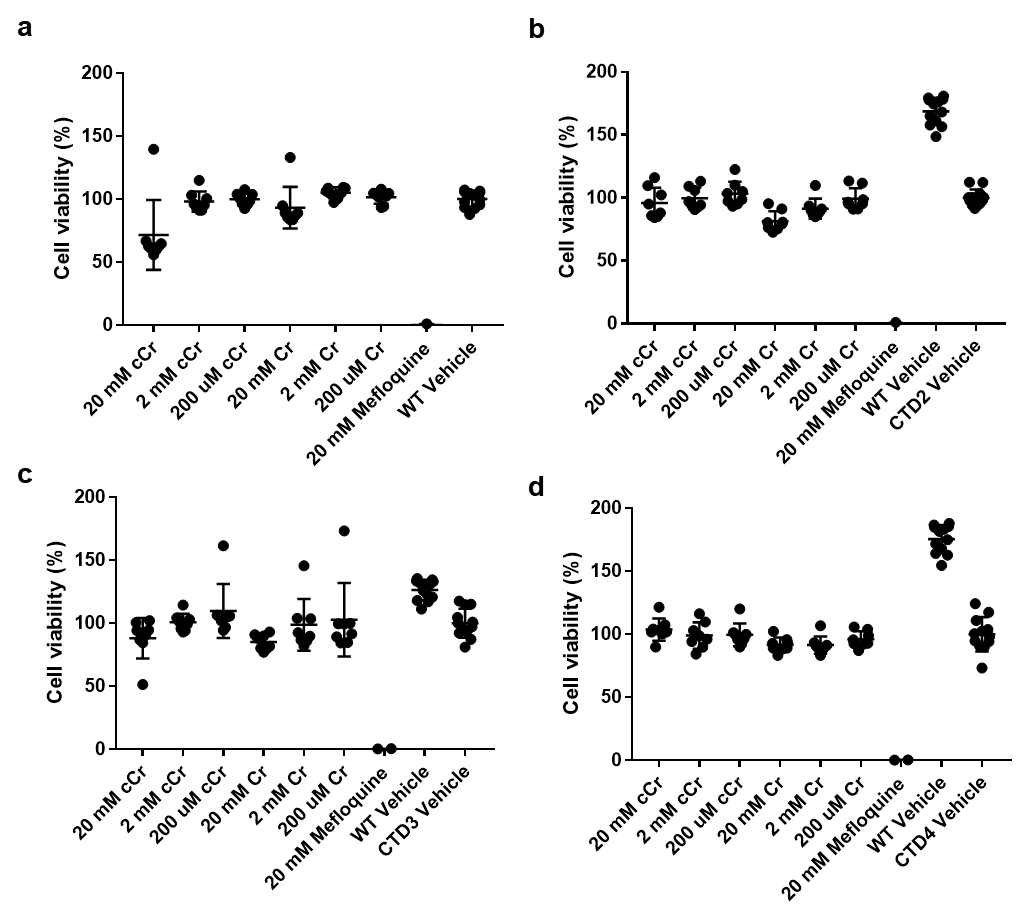


Supplementary Figure 1. Cytotoxicity of cCr and Cr-d3 in WT and CTD patient fibroblasts. ATPlite was used to measure the cytotoxic effects of 20 mM, 2 mM, and 200 µM Cr-d3 or cCr, vehicle, and 20 mM mefloquine for 72 hours in **(a)** WT, **(b)** CTD patient line 2, **(c)** CTD patient line 3, **(d)** CTD patient line 4 fibroblasts. Luminescence values normalized to WT vehicle in a, or CTD vehicle in b-d, shown on the y-axis. Data shown as mean ± S.D. n≥4 technical replicates.
