# Supplementary figures and images for: Phosphocyclocreatine is the dominant form of cyclocreatine in control and creatine transporter deficiency patient fibroblasts
Source: Pharmacol Res Perspect. 2019 Dec 20;7(6):e00525. doi: 10.1002/prp2.525 (PMC6924099; doi:10.1002/prp2.525)

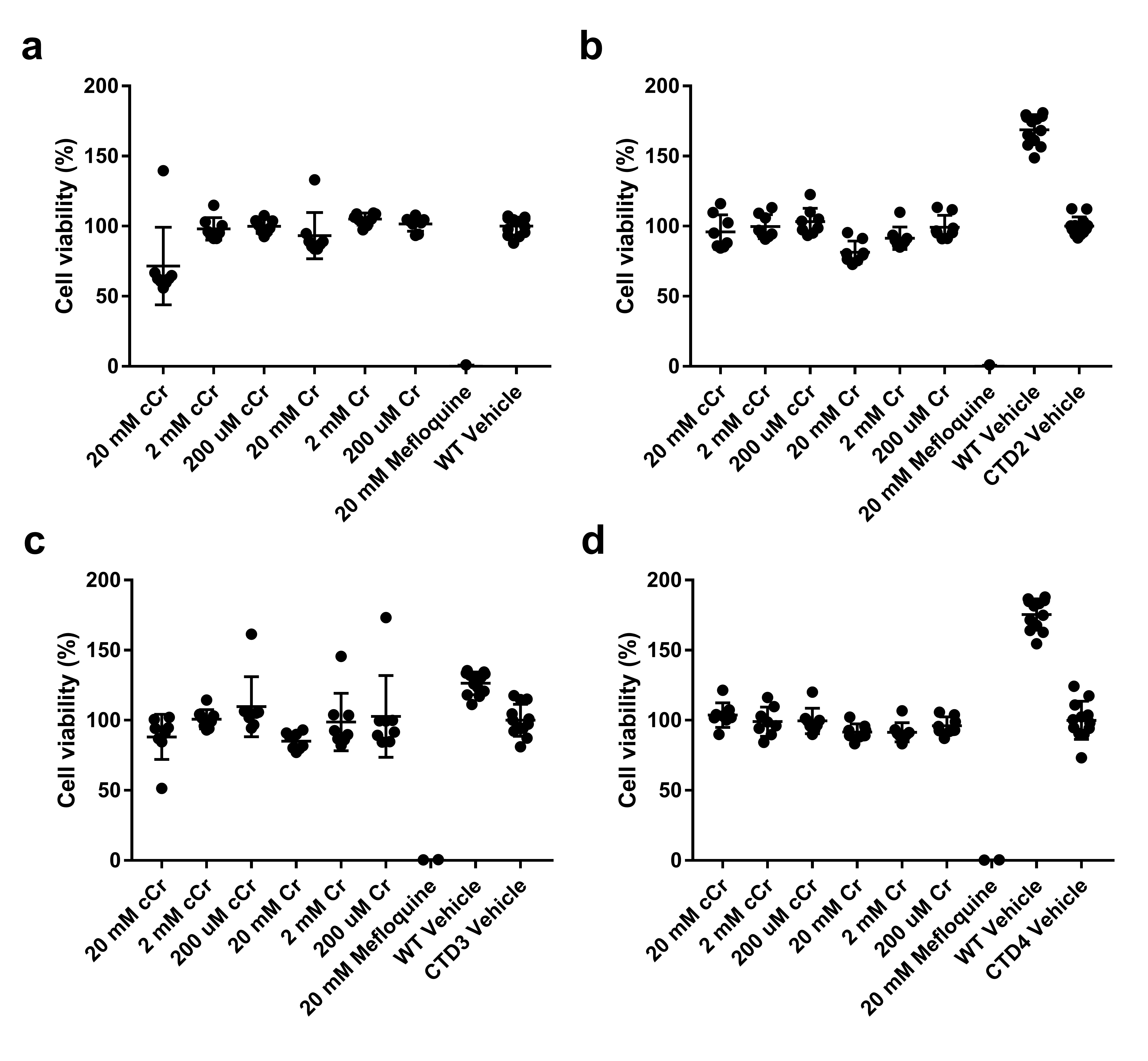

Supplement: Supplementary file 2 [file PRP2-7-e00525-s002.tif]
